# “NiCo Buster”: engineering *E. coli* for fast and efficient capture of cobalt and nickel

**DOI:** 10.1186/1754-1611-8-19

**Published:** 2014-08-01

**Authors:** Alexandre Duprey, Viviane Chansavang, Franck Frémion, Clémence Gonthier, Yoann Louis, Philippe Lejeune, Fanny Springer, Valérie Desjardin, Agnès Rodrigue, Corinne Dorel

**Affiliations:** 1 iGEM team INSA Lyon, Plateforme de Biologie de Synthèse, Département Biosciences, INSA Lyon, 69621 Villeurbanne Cedex, France; 2 Université de Lyon, INSA-Lyon, LGCIE, Villeurbanne F-69621, France; 3 Université de Lyon, INSA-Lyon, Université Claude Bernard Lyon I, CNRS, MAP, UMR5240, Villeurbanne F-69621, France

**Keywords:** Bioremediation, Cobalt, Nickel, Biofilm, Biofilter, NiCoT, Synthetic biology

## Abstract

**Background:**

Metal contamination is widespread and results from natural geogenic and constantly increasing anthropogenic sources (mainly mining and extraction activities, electroplating, battery and steel manufacturing or metal finishing). Consequently, there is a growing need for methods to detoxify polluted ecosystems. Industrial wastewater, surface water and ground water need to be decontaminated to alleviate the contamination of soils and sediments and, ultimately, the human food chain. In nuclear power plants, radioactive metals are produced; these metals need to be removed from effluents before they are released into the environment, not only for pollution prevention but also for waste minimization. Many physicochemical methods have been developed for metal removal from aqueous solutions, including chemical coagulation, adsorption, extraction, ion exchange and membrane separation; however, these methods are generally not metal selective. Bacteria, because they contain metal transporters, provide a potentially competitive alternative to the current use of expensive and high-volume ion-exchange resins.

**Results:**

The feasibility of using bacterial biofilters as efficient tools for nickel and cobalt ions specific remediation was investigated. Among the factors susceptible to genetic modification in *Escherichia coli*, specific efflux and sequestration systems were engineered to improve its metal sequestration abilities. Genomic suppression of the RcnA nickel (Ni) and cobalt (Co) efflux system was combined with the plasmid-controlled expression of a genetically improved version of a specific metallic transporter, NiCoT, which originates from *Novosphingobium aromaticivorans.* The resulting strain exhibited enhanced nickel (II) and cobalt (II) uptake, with a maximum metal ion accumulation of 6 mg/g bacterial dry weight during 10 min of treatment. A synthetic adherence operon was successfully introduced into the plasmid carrying the improved NiCoT transporter, conferring the ability to form thick biofilm structures, especially when exposed to nickel and cobalt metallic compounds.

**Conclusions:**

This study demonstrates the efficient use of genetic engineering to increase metal sequestration and biofilm formation by *E. coli*. This method allows Co and Ni contaminants to be sequestered while spatially confining the bacteria to an abiotic support. Biofiltration of nickel (II) and cobalt (II) by immobilized cells is therefore a promising option for treating these contaminants at an industrial scale.

## Background

Metals are commonly used by industry but are generally harmful for the environment [1]. Consequently, liquid effluents need to be decontaminated, not only when heavy metal concentrations are high but also when concentrations are low to avoid bioaccumulation because these substances display long-term persistence in sediments. Chemical and physical methods (for example, chemical precipitation and ionic exchange resins) can be ineffective or extremely expensive when the metals ions are present at micromolar concentrations [2]. In the context of nuclear power plants, radioactive hazard adds to the natural toxicity of the metals used; thus, their removal from effluents is critical. Among the metals released by the types of steel alloy used in pipes, two are of particular importance: (i) nickel, which is present in low concentrations (approximately 1 mM) [3] and (ii) cobalt, notably its activated radioisotope ^60^Co, which is present in trace quantities (approximately 20 nM [4]) but is particularly harmful due to its long half-life (5.27 years) and emission of high energy γ rays (1.17 and 1.33 MeV) [3]. Current methods of removing these metals ions involve the use of ion-exchange resins, which, although efficient, are non-selective, expensive and require the use of high volumes to decontaminate metals at low concentrations, especially cobalt, resulting in high treatment costs [5]. The removal and concentration of these metals is achieved using large volumes of resin, which then have to be treated as radioactive waste involving long-term storage in dedicated centers until the radioisotopes have decayed. Thus, the need for space is critical in radioactive waste storage, and demand is high for cheaper, more specific solutions that reduce the volumes involved.

For decades, the use of biological tools has been suggested as an efficient and cost-effective solution for metal remediation [6]. Non-living biomass biosorbents (algae, fungal, yeast or bacterial biomass) can be used to immobilize heavy metals with low selectivity [2,4,7]. Engineered and non-engineered bacteria (e.g., *Escherichia coli*, *Deinococcus radiodurans, Citrobacter sp.*) have been tested for use in the remediation of metals such as mercury [8] and uranium [9]. Indeed, bacteria grow rapidly in liquid media, can use a wide variety of nutrients and have developed many efficient mechanisms to resist and detoxify harmful metals [10]. Some bacteria can live under extreme conditions, such as *D. radiodurans,* which can grow in the presence of 60 Gy/h; thus, this organism exhibits potential for use in treating liquid radioactive waste. Another important issue in bioremediation is the recovery of the biosorbent that contains the heavy metals. Developing a process that minimizes costs while efficiently separating solids from liquid is challenging. Flocculent *S. cerevisiae* cells and calcium alginate beads containing *D. radiodurans* bind metals ions efficiently and can be removed easily from the treated solution [7,11]. Magnetic chitosan, a modified biopolymer, has been used successfully to remove Cu(II), Co(II) and Ni(II) ions from aqueous solutions; however, this approach requires multi-step extraction and modification processes to prepare the chelating resin [12].

It has been extensively documented that some species of bacteria contain dedicated transporters that favor the uptake of Ni(II) and Co(II). These transporters include the *nikABCDE* system found in *E. coli*[13], the NiCoT family found in eubacteria, archaea and fungi [14] and some ABC transporters [15,16]. Using genetic techniques, it has been proven that such transporters, which differ in their specificity towards Co(II) and Ni(II) [15,17,18], can be expressed in various bacterial species [17,19]. However, the metal accumulation properties of these bacteria are counterbalanced by the presence of efflux pumps, which expel these metals ions out of the cell. The *rcnA* system is the only known Ni(II) and Co(II) efflux pump in *E. coli*[20]. The genes encoding these efflux pumps can be disabled, leaving the imported metals trapped inside the cytoplasm and unable to exit the cell membranes.

Based on these two principles, a Co-accumulating *E. coli* strain has been engineered [3]; however, this strain had a major disadvantage: bacteria could not be recovered easily from the decontaminated effluent. However, several bacteria have the ability to attach strongly to surfaces using various polymers, thereby forming biofilms. Among these polymers are *E. coli* curlis, adhesion factors that belong to the amyloid fiber family [21]. Curlis mediate adhesion to a wide range of biological or inert surfaces [22]. This study focused on whether the adherence of a metal-accumulating bacterium could be enhanced to form a functional biofilter.

For this purpose, we engineered the previously described Ni- and Co- accumulating bacteria [3] by adding a metal-inducible curli overproduction system [22]. The adherence and the metal accumulation capacities of the engineered strain were assessed using various qualitative and quantitative methods. The increased ability of the engineered strain to stick to inert surfaces facilitates its immobilization on a solid support and its removal from the decontaminated effluents. Hence, this strain might be a promising candidate for use in an industrial-scale biofilter.

## Results and discussion

### Conception and design of the engineered strain

Our rational design of the Ni/Co Buster strain was aimed at modifying three cell functionalities. First, a constitutive fluorescent version of the MG1655 strain (SCC1, referred to as S29 in this manuscript) was used as a basic chassis to facilitate the monitoring of bacterial dispersion and biomass formation. Then, Ni/Co(II) capture optimized at two levels: preventing metal efflux and increasing metal uptake, as described below. Wild-type *E. coli* contains a Co and Ni efflux system, called Rcn (Ni and Co Resistance); this system is present inside the MC4100 strain, preventing the accumulation of Co(II) and Ni(II) [16]. As described in the Methods section, this system was disabled by transferring a *rcnA::uidA*-*kan* cassette to the fluorescent MG1655 strain S29 by P1 transduction. As expected, the resulting *rcnA* strain (S48) showed increased sensitivity to the toxic effects of Ni(II) and Co(II) compared to the parental strain. This result indicates that the P1 transduction procedure successfully inactivated the *rcnA* gene (data not shown). To further improve the performance of this Co- and Ni-accumulating strain, a genetic device was designed that allowing enhanced uptake of these metals. A synthetic metal uptake gene encoding the Ni(II) and Co(II) transporter (NiCoT) from *Novosphingobium aromaticivorans,* which is optimized for expression in *E. coli,* was placed under the control of the strong promoter *Ptac*, as described in the Methods section. This transporter was chosen due to its outstanding Co(II) accumulation and average Ni(II) accumulation capacities compared to other transporters of the NiCoT family [14].

**Figure 1:**
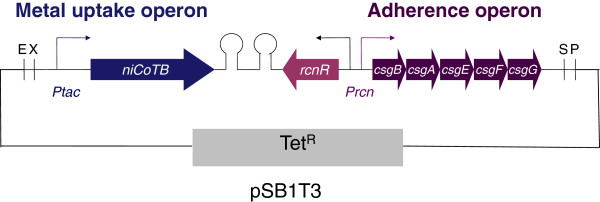
**Schematic representation of the engineered plasmid conferring constitutive metal uptake and inducible adherence.** Two synthetic operons were designed and introduced in the pSB1T3 plasmid to generate pIG50 (Table 1). The first operon controls metal uptake by placing the codon-optimized nickel and cobalt transporter gene from *Novosphingobium aromaticivorans nicoTB* under the control of the strong promoter *Ptac*. The second operon confers metal-inducible adherence due to genes encoding curli structural (CsgA, CsgB) and assembly (CsgE, CsgF, CsgG) proteins under the control of the nickel and cobalt sensitive promoter from *E. coli*, *Prcn*. The bidirectional terminator Bba_B0014 [37] was added to prevent read-through transcription *Ptac*, *nicoTB*-BBa_B0014 and *rcn*-*csgBAEFG* were obtained separately by direct synthesis and were assembled in the high-copy vector pSB1T3.

Finally, cell adherence was enhanced by implementing synthetic adherent curli machinery (carried by pIG2 [18]) to prevent cell dispersion in the treated effluent using the P*rcn* bidirectional promoter. In *E. coli*, this promotor controls the transcription of *rcnR* in one direction and controls the transcription of *rcnAB* in the opposite direction. Between the transcriptional start sites of *rcnR* and *rcnAB*, there are two binding sequences for RcnR, a repressor that controls its own expression as well as the expression of *rcnAB* in response to intracellular concentrations of Ni(II) or Co(II) [23]. In this construct, the bidirectional promoter *Prcn* controls the curli operons *csgBAEFG* in the forward direction and the gene encoding the cognate metallo-regulator RcnR in the opposite direction (Figure 1). This design allows reinforced bacterial adherence in the presence of Ni(II) and Co(II) but is not expected to provide an absolute adherence control [18]. In this work, *Ptac*-*nicoTB* was cloned upstream of the *Prcn-csgBAEFG* construct. Readthrough was prevented by introducing a bidirectional terminator between these regions. The resulting pIG50 plasmid, which is described in Figure 1, was transformed into the fluorescent *rcnA* strain S48 to create the Ni/Co Buster strain, which is also referred to as S61 or the “engineered strain” in this paper.

### The pIG50 plasmid confers enhanced adherence of the MG1655 *rcnA* mutant to polystyrene

**Figure 2:**
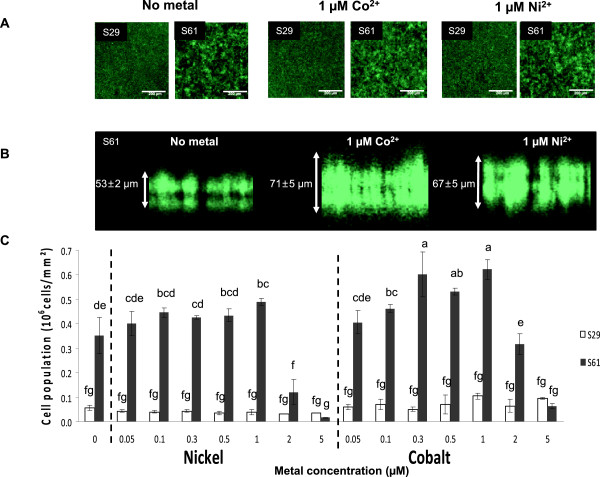
**The pIG50 plasmid enhances MG1655 adherence to polystyrene.** The GFP-tagged MG1655 strain (S29) and its engineered version were transformed with pIG50 (S61) and incubated in 96-well plates for 24 h at 30°C in M63G medium supplemented with various concentrations of metal ranging from 0.05 to 5 μM as indicated in the figure. The efficiency of biofilm formation was estimated based on microscopic observation or the fluorescence of the attached cells as described in the Methods section. **A)** Fluorescence microscopy. Biofilms formed by the S29 and S61 strains in the presence or absence of 1 μM Ni and 1 μM Co were observed. A sharp increase of adherence resulting from transformation with the pIG50 plasmid was observed. This phenomenon is quantified in Figure 2C. **B)** Confocal laser scanning microscopy. The thickness of the biofilm formed by the S61 strain in the presence or absence of cobalt and nickel was estimated using confocal microscopy to illustrate the slight increase in adherence conferred by the presence of metal. Five fields were observed per strain, and three measurements of biofilm thickness were performed per field. The biofilms of the engineered strains were significantly thicker in the presence of metal (p < 0.005, Dunnett’s test). **C)** Spectrofluorimetry. The effect of metal concentration on S61 biofilm formation was estimated based on the fluorescence intensity of the attached cells. The fluorescence intensity of the S61 biofilms was converted to the corresponding number of cells/mm^2^ based on a standard curve as described in the Methods section section and compared to the reference values obtained from biofilms formed under the same conditions by the parental S29 strain. Data represent the mean of 3 replicates (=3 wells), and error bars represent standard deviations. Significant differences are indicated using lowercase letters, and different letters indicate significant differences (Tukey’s test, p < 0.05).

Fluorescence and confocal laser scanning microscopy (CSLM) and spectrofluorimetry measurements were used to verify increased adherence of the engineered bacterial strain compared to controls. First, biofilm formation of the GFP-tagged S29 and S61 strains was observed in the presence or absence of 1 μM Ni(II) or Co(II) under a microscope after 5, 15, 24 and 48 h of culture in 96-well polystyrene plates. The behavior of the S29 and S61 strains differed after only 5 hours of incubation at 30°C. Whereas the adherent wild-type cells tended to remain distinct in the control samples without metal, the engineered strain formed clusters (data not shown). The S61 cell clusters evolved a dense biofilm after 24 hours of incubation, whereas the S29 strain failed to develop dense and structured biofilms (Figure 2A). By comparing strains carrying or not carrying the pIG50 plasmid, we observed that the plasmid impaired bacterial growth. The average doubling time of the transformed cells increased from 256 ± 20 min to 300 ± 17 min (n = 7, *t*-test, p < 0,001). Although the S29 strain grew better, it did not have the capacity to form a thick biofilm, highlighting the enhanced adherence properties of the pIG50-harboring strain. These results show that the pIG50 plasmid confers increased adherence to polystyrene on the host-cells, even in the absence of metal. In the presence of Ni(II) or Co(II), an additional slight increase in surface occupancy by the engineered strain was observed (Figure 2A). The increased production of biofilm in response to metals was confirmed using a confocal laser-scanning microscope; thicker biofilms were formed by the engineered strain S61 in the presence of metal (Figure 2B).

To obtain a more accurate view of this phenomenon, the autofluorescent S29 and S61 strains were grown in a 96-well plate in the presence of a wide range of Ni(II) and Co(II) concentrations (7 concentrations ranging from 0.05 to 5 μM of metal ions). After 24 h of culture at 30°C, the supernatant was thoroughly removed, and the fluorescence of the remaining biofilm was measured in each well using a fluorimeter as described in the Methods section. Due to their genomic insertions (Table 1), the S29 and S61 strains constitutively produce GFP. The fluorescence of such strains is therefore directly proportional to the number of cells in the two fractions (i.e., in the supernatant and the biofilm); therefore, the fluorescence can be used to estimate the ratio of adherent cells. A standard curve was established to facilitate conversion between units of fluorescence and biomass as described in the Methods section. For all non-toxic metal concentrations (*i.e*., concentrations of less than 2 μM Ni(II) or Co(II)), the biofilms formed by the engineered strain hosted significantly more cells than the parental strain (compare S61 and S29, Figure 2C). In agreement with the observations performed using fluorescence microscopy (Figure 2A) and CSLM (Figure 2B), a slight increase in biofilm formation was detected in the presence of metals by directly measuring cell fluorescence after separating the free and sessile bacteria. No significant change was observed after 48 h of culture (data not shown).

**Table 1:** Bacterial strains and plasmids used in this study

**Strain**	**Relevant description**	**Reference**
ARY023	*rcnA*::*uidA-kan*	[16]
HYD720	Δ*nikA-kan*	[24]
MG1655	F- λ-	
S57	MG1655/pIG50	this study
S59	MG1655 *nikA*	this study
S63	S59/pIG50	this study
SCC1 = S29	MG1655 (PA1/04/03 gfpmut3*Cm)	[25]
S48	MG1655 (PA1/04/03 gfpmut3*Cm) *rcnA::uidA-kan*,	this study
S61 “NiCo buster”	S48/pIG50	this study
1137 = S71	MG1655 *malT*::*Tn*10 *ompR234 csgA*::*uidA-kan*	[26]
**Plasmids**	**Relevant description**	**Reference**
pSB1C3	pUC19-derived pMB1 (copy number: 100–300) Cm^R^	[27]
pSB1T3	pUC19-derived pMB1 (copy number: 100–300) Tet^R^	[28]
pIG2	*rcn-csgBAEFG* inserted at sites *Eco*RI/*Pst*I of pUC57	[22]
pIG49	*Ptac*-*nicoTB* in pSB1C3	this study
pIG50	*Ptac*-*nicoTB*-*rcn*-*csgBAEFG* in pSB1T3	this study

Taken together, these results show that the engineered strain S61 constitutively forms biofilms and that the thickness of these biofilms is moderately but significantly enhanced in the presence of Ni(II) or Co(II). This outcome is consistent with previous experiments showing that the *rcn* promoter is leaky [29]. The Prcn promoter was preferred over the curli promoter to reduce the complexity of the genetic regulation of curli-mediated adherence. The curli endogenous promoter is not only regulated by Ni(II) but is one of the most complex promoters in *E. coli*[26,30] and is affected by a wide range of physiochemical signals, including temperature and osmolarity, and numerous regulators are known to modulate its expression [31,32]. Prcn is more stable with respect to environmental modification [23,29] and increases the ability of cells to bind to their support in the presence of these metals. Both of these properties are expected to make the engineered strain more attractive for industrial application.

Cell immobilization is important for bioremediation for three reasons: it increases resistance to pollutants, confines the bacteria, and facilitates removal from the water phase, thereby facilitating the recovery of pollutant metals (reviewed in [33]). Immobilization can be achieved by adsorption, or by entrapment in a polymer network such as an alginate (methods reviewed in [24]). Intrinsic entrapment in a biofilm matrix, as designed and realized in this work, is a promising solution that limits the costs associated with immobilization.

### Efficiency of the engineered metal uptake transporter

The specific uptake of Ni(II) or Co(II) was catalyzed by the Ni/Co uptake transporter from *Novopshingobium aromaticivorans*. This transporter is a single permease that belongs to the class II family of NiCoT transporters [10] and has been previously been shown to import both Ni(II) and Co(II), although Co(II) is imported with higher efficiency [2,10]. In the engineered strain, the amino acid sequence of the NiCoT transporter was optimized for expression in *E. coli* (see methods)*.*

**Figure 3:**
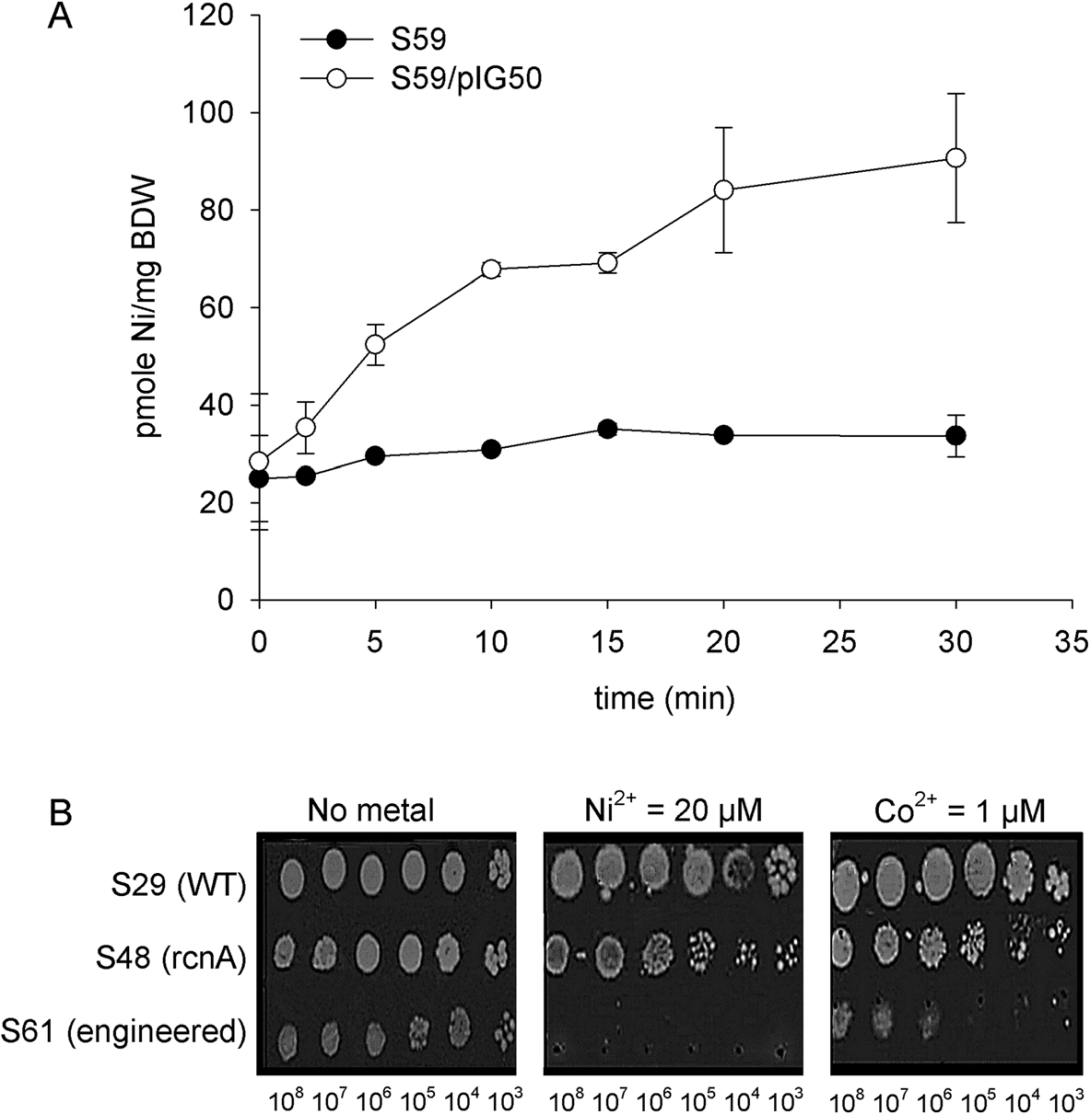
**Functionality of the NiCoT transporter in the engineered strain. A)** Uptake of nickel by the engineered transporter. The strains S59 (MG1655 *nikA*) and S63 (S59/pIG50) were cultured in LB medium to an OD_600_ of 0.6, at which time 150 nM ^63^NiCl_2_ was added. The cells were recovered after 0, 2.5, 5, 10, 15, 20 and 30 minutes of contact by filtration using a nitrocellulose filter. The captured nickel was estimated by measuring the radioactivity of the filter. Data represent the mean of 2 replicates, and error bars represent standard deviations. **B)** Cell viability tests. The sensitivities of wild type (S29), *rcnA* mutant (S48) and engineered (S61) strains towards nickel and cobalt were compared. Five microliters of cells at the indicated concentrations were spotted onto M63G (supplemented or not supplemented with metal) and incubated at 37°C for 48 h. Metal concentrations ranging from 1 μM to 50 μM were tested. Only one concentration (indicated below the image) is shown, corresponding to the MIC of strain S61. In the presence of 20 μM Ni(II), the engineered strain exhibited no growth; in contrast, the growth of the parental strain was not affected, and the growth of the *rcnA* chassis was slightly decreased.

The efficiency of the NiCoT transporter was characterized using a quantitative Ni(II) uptake assay. In *E. coli*, Ni(II) uptake is mediated by the *nikABCDE* system [13]; in contrast, no Co-specific uptake system has been described thus far. To measure the specific uptake of Ni(II), experiments were carried out in a *nikA* mutant strain in the presence of a ten-fold excess of Mg ions to avoid nonspecific Ni(II) uptake via MgtA or CorA Mg transporters [13]. Moreover, the cells were washed with EDTA before radioactivity counting to prevent nonspecific Ni-binding on the bacterial cell wall. The accumulation of Ni(II) was monitored in a time course assay (30 min) by incubating the cells with ^63^Ni. The intracellular concentration of ^63^Ni per milligram of bacterial dry weight was then determined as described in the Methods section. Figure 3A shows that the accumulation of intracellular nickel by the engineered S63 strain begins during the first minutes of contact with the metal. In contrast, the parental *nikA* strain S59 exhibited little, if any, accumulation of ^63^Ni. In the presence of 150 nM of radioactive metal, specific ^63^Ni uptake by S59/pIG50 appears to attain equilibrium after 30 minutes, reaching 6 μg/g of BDW (Figure 3A). This result shows that the pIG50 plasmid carrying the *nicoT* codon-optimized construct allows rapid and significant Ni(II) accumulation. Therefore, the engineered high-affinity transporter NiCoT from *N. aromaticivorans* appears to be fully functional in *E. coli*.

The physiological effects of metal uptake by the engineered strain were then investigated. Considering that an intracellular accumulation of metal would increase the sensitivity of the strain to metal [20], we assessed the sensitivity of the engineered strain towards Ni(II) and Co(II). In the absence of metal, the growth of the engineered strain was comparable to that of the S29 parental strain or the S48 *rcnA* chassis (Figure 3B). In contrast, distinct phenotypes are observed for the three strains in the presence of 20 μM Ni(II) (a subinhibitory concentration). Whereas the growth of the parental strain was not affected and the growth of the *rcnA* strain was slightly affected by nickel, a dramatic loss of viability of the engineered strain occurred in the presence of the metal, indicating metal poisoning. Similar results were obtained in the presence of Co(II) at a lower concentration (1 μM). These results are consistent with previous studies that have shown that Co(II) is toxic at lower concentrations than Ni(II) [20,29]. This increase in Co(II) and Ni(II) sensitivity further demonstrates the functionality of the NiCoT transporter.

### Capture of nickel and cobalt by the engineered “NiCo buster” strain

**Figure 4:**
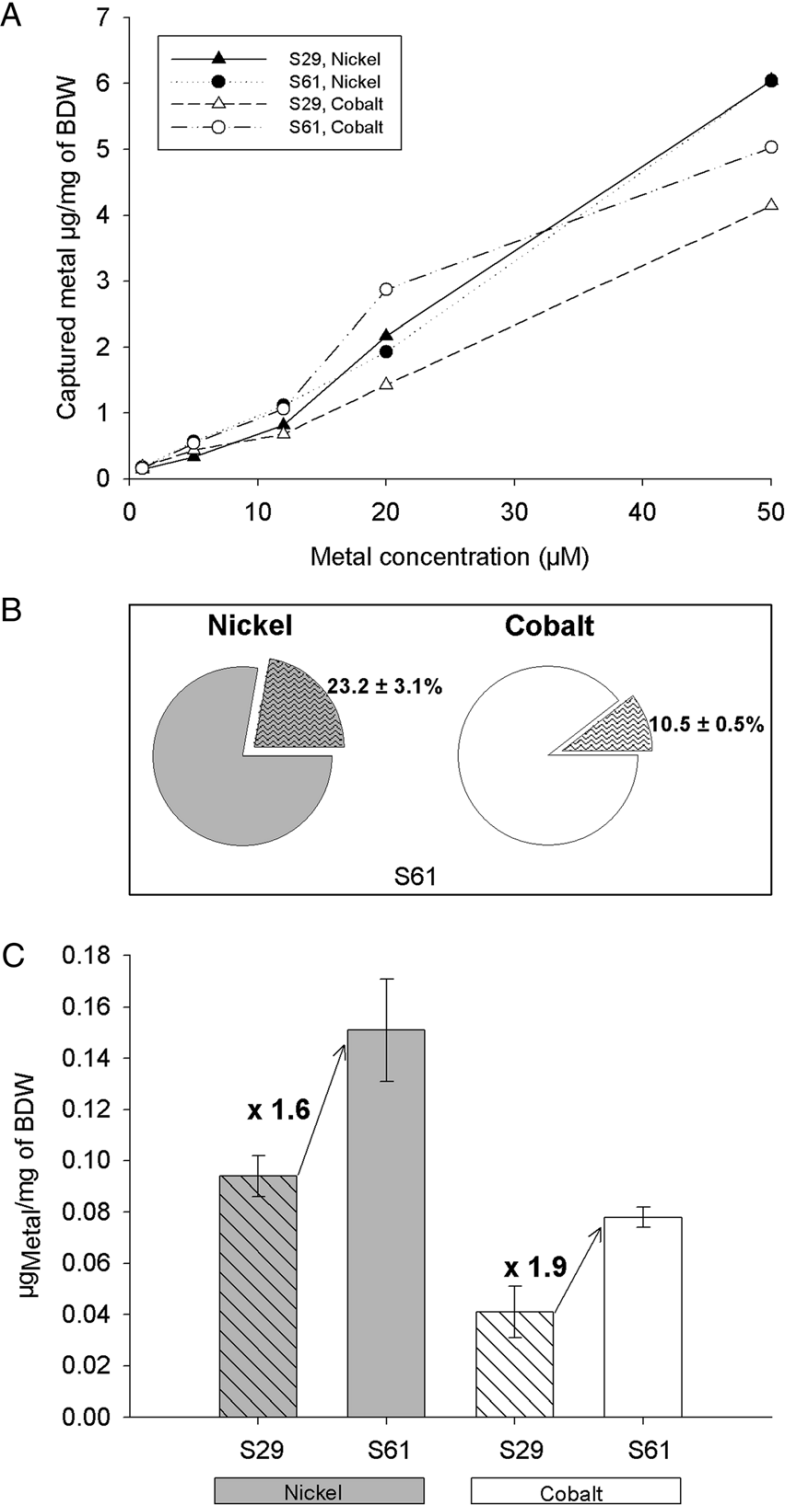
**Capture of nickel and cobalt by the “NiCo buster” strain, as measured using ICP-MS.** The S61 and S29 strains were cultured for 24 hours in M63G supplemented with the appropriate antibiotic at 30°C in Petri dishes. The medium was then removed and replaced by a metal solution prepared in sterile water. After 10 min (Figure 4A) or 30 min (Figure 4B and C) of contact between the bacterial biofilms and the metallic solution, the supernatant was discarded. The biofilm was collected, acidified and mixed with rhodium as an internal standard as described in the Methods section. The metal concentration of each sample was assayed using the ICP-MS technique as described in the Methods section. **A)** Absolute metal capture yields, expressed as the mass of captured metal divided by the mass of the biofilm. **B)** Relative contribution of non-specific binding and specific internalization. Absolute metal internalization yields, expressed as the mass of captured metal after washing the cells with EDTA (broken lines) divided by the mass of captured metal without washing the cells with EDTA (plain colors) after incubation in the presence of Ni (grey) or Co (white) for the S61 strain. The values shown are the means of three independent replicates. **C)** Contribution of the specific internalization provided by the engineered strain. The S29 strain (dashed lines) or S61 strain (plain color) was incubated in the presence of Ni (grey) or Co (white). The values represent the amount of metals assayed inside the cells in three independent samples after treatment with EDTA.

Having verified that the adherence operon and the metal uptake operon were both functional, we assessed the metal-accumulating capacities of the engineered “NiCo buster” (S61) biofilm. Because the S61 strain was engineered by deleting *rcnA* and adding pIG50, the efficiency of the engineered strain was measured against a strain possessing neither modifications, i.e., the wild-type (S29) strain. The engineered (S61) and parental (S29) strains were grown for 24 h in Petri dishes. After free-floating cells were discarded, the adherent cells were incubated for 10 min in the presence of increasing amounts of metal ions (5, 12, 20 and 50 μM of Ni(II) or Co(II)). The efficiency of metal capture was then quantified using ICP-MS (Inductively Coupled Plasma Mass Spectrometry). For both metals and both strains, cellular sequestration increased with metal concentration. S61 appeared to accumulate slightly more metal than S29 (Figure 4A) but only within limited range of the tested concentrations; this was especially true for Ni(II). The concentration of captured metals did not reach a plateau in the tested range (0–50 μM). These results, together with the ^63^Ni uptake results presented in Figure 3A suggest that longer contact time might be required to reach the full metal capture potential of the bacterial cells. Moreover, the similar behaviors of the metal sequestration capacities provided by the two strains suggest that the total metal binding capacity of the bacteria arise from both specific and non-specific binding events*.* Indeed, for both strains, the total metal sequestration increased with external metal concentration. This might be due to nonspecific binding to the bacterial cell surface [17].

To evaluate the fraction of metal ions that bound nonspecifically to extracellular structures and the contribution of the NiCoT transporter, experiments like those presented in Figure 4A were performed. In this set of experiments, a single metal concentration was used (10 μM), and the contact time between the bacteria and the metal was 30 min to enable equilibrium to be reached. The experiment was repeated using three independent biological replicates. Half of the bacteria were subjected to ICP measurements without any previous treatment, and the remaining half was washed with EDTA before being titrated (see the Methods section). The treatment of intact bacteria with EDTA should remove most of the extracellularly bound metal. Comparing the EDTA-washed and unwashed fractions of the S61 strain showed that 80% of the captured Ni(II) or 90% of the captured Co(II) are bound by the extracellular matrix (Figure 4B). The comparison between the EDTA-washed WT strain (S29) and the EDTA-washed engineered strain (S61) should demonstrate the contribution of the NiCoT transporter and the absence of the RcnA efflux pump. In the experiment, the S61 strain accumulated 1.6-fold more Ni(II) than did the WT strain, and the S61 strain accumulated 1.9-fold more Co(II) than did the WT strain (Figure 4C). These results are consistent with those shown in Figure 4A and clearly show the importance of the NiCoT transporter to the capture of the metals.

In conclusion, the *E. coli* bacteria accumulated 4.8 to 6 mg/g dry weight in only 10 minutes when the cells were exposed to 50 μM Ni(II) or 50 μM Co(II), respectively, and accumulated 1.8 to 3 mg/g of bacterial dry weight when exposed to 20 μM Ni(II) or 20 μM Co(II), respectively. The performance obtained was better than that achieved using transgenic tobacco (*Nicotiana tabacum*) expressing a bacterial NiCoT; in that experiment, the tobacco accumulated 0.6 to 1.5 mg/g of plant dry weight in 30 days when the pre-cultivated, hydroponically grown plants were exposed to 20 μM Ni(II) or 20 μM Co(II), respectively [34]. Metal hyperaccumulator plants, such as *Thlaspi caerulescens*, have been reported to accumulate 2.8 mg of Ni/g of whole plant dry weight in hydroponic culture when grown for 3 weeks in presence of 10 μM metal [21,22] (representing slightly better performance, which was achieved at the cost of strongly increased decontamination times). Direct decontamination of wastewater containing low concentrations of non-radioactive Ni(II) and Co(II) might be achieved more rapidly and at lower cost using our engineered bacteria rather than plants that require weeks to grow. Highly contaminated effluents (e.g., from mining and electroplating industries) could first be treated using conventional chemical or physical methods or using new biopolymer biosorbents [7,12]. Bacteria could then be used to polish the wastewater in an additional treatment step to attain very low heavy metal ions concentrations that would comply with increasingly restrictive laws that regulate the maximum acceptable concentrations of metal in water. Our results show that Ni(II) and Co (II) metals are mainly captured by non-specific binding mechanisms. The NiCo Buster design, however, allows to increase the metal internalization by a factor of 1.6 (Ni) to 1.9 (Co), compared to the parental strain. This is of special interest when treating complex effluents, which often contain large amounts of iron and traces of other divalent cations.

The engineered *E. coli* “Co/Ni Buster” strain appears therefore as a promising candidate for the depollution and retrieval of these metals in radioactive effluents due to the rapidity of its action, achieving comparable results to existing systems with at least a 2000-fold reduction of incubation time. In processes using our immobilized strain, the amount (volume) of material necessary to remove radioactive Ni(II) and Co(II) material should be less important, and the removal of these two metals could have a positive impact on the classification of these radioactive wastes. Furthermore, in the future, our genetic construct could be transferred to other organisms, such as *Deinococcus radiodurans*, a very radioresistant strain in which genes from *E. coli* can be successfully expressed [35]. The NiCo Buster system would however require optimization before industrial usage. One limitation is the presence of antibiotic resistant genes in the engineered strain. This raises the possibility for horizontal transfer antibiotic resistance genes from the engineered strain to environmental bacteria. Genome editing would permit to integrate all the exogenous sequences in the chromosome and remove the superfluous sequences.

## Conclusions

The two most important issues related to metallic waste processing are 1) reducing the environmental degradation resulting from disposal and 2) recycling the metals of economic interest, such as Co(II) and Ni(II). Due to the high cost of conventional physicochemical methods, microorganisms and plants have been already achieved wide application, for example, in sewage treatment. Successful uses of microbial bioremediation have been reviewed in [36,37]. Our study shows that the development of synthetic biology may play a role in the improvement of bioremediation processes, especially regarding their time requirements. Another important issue is that bacteria can be immobilized on solid supports to allow easy removal of “metal-loaded” bacteria from the bioreactor. This issue was addressed here by enhancing the natural adhesiveness of *E. coli* in response to the presence of the metal to be refined. If not total, adherence was significantly improved in our synthetic construct, thus paving the way for future developments. Concerning the metal-binding capacities of the strains, we showed here that most of the bound metal was present in the outer envelope. Nevertheless, the presence of the NiCoT transporter enhanced metal internalization by a factor of 1.6 to 1.9. This is of special interest when treating complex contaminated effluents, which often contain large amounts of iron and traces of other divalent cations. We showed here that the tested bacterial cells act as a non-specific metal sponge and that the transporter is able to selectively uptake trace metals.

In brief, genetically improved metal accumulation and adherence were successfully implemented, as demonstrated in this work. We suggest that the “Ni/Co Buster” strain could be used to create a new generation of biofilters that are designed for the remediation of Ni(II) and Co(II).

## Methods

### Bacterial strains and media

The *E. coli* K-12 MG1655 derivatives used in this study are described in Table 1. The bacteria were grown in either LB (Luria-Bertani [38]) or M63G (M63 [38], supplemented with glucose 0.2% and LB 1:100 v/v) media. When appropriate, Co(II) or Ni(II) were added to the media as CoCl_2_ or NiSO_4_, respectively. The antibiotics chloramphenicol (20 μg/mL), kanamycin (50 μg/mL) and tetracycline (10 μg/mL) were purchased from Sigma and used in the experiments. The media were inoculated at a final bacterial concentration of 10^7^ cells/mL. Stock metal solutions were prepared in sterile water. Short incubations of biofilm with metal (less than 30 min) were carried out using metal solutions diluted in water.

### Genetic methods

Phage P1 transductions of strains SCC1 and MG1655 were carried out as described by Miller [38]. Strains ARY023 [20] and HYD720 [39] were used as donors for *rcnA::uidA*-*kan* and *ΔnikA-kan cassettes,* respectively. *rcnA::uidA*-*kan* was transduced in strain SCC1 to generate strain S48. *ΔnikA-kan* was transduced in strain MG1655 to construct strain S59. Transformations were performed using the TSS transformation protocol described in [40]. All synthesized genes were obtained from Genecust Europe, Luxembourg. Enzymes and buffers were supplied by Fermentas (Germany), and enzymatic reactions were performed according to the supplier’s instructions. All BioBrick assemblies were performed using the RFC10 standard. BioBrick assemblies used either the standard or the 3A assembly method [41]. Briefly, standard assembly is performed by digesting the insert using *XbaI* and *PstI*, digesting the vector using *SpeI* and *PstI*, and then ligating both. 3A assembly is performed by digesting the upstream insert using *EcoRI* and *SpeI,* digesting the downstream insert using *XbaI* and *PstI*, digesting the vector using *EcoRI* and *PstI*, and ligating the products.

### Construction *of Ptac*-*nicoTB* and construction of the engineered plasmid pIG50

*Ptac* and *nicoTB* were obtained by direct synthesis. *Ptac* uses the promoter sequence found in the plasmid pKK233-2 (Clontech). *nicoTB* comprises a strong RBS [42], the *nicoT* coding sequence found at locus Saro_0344 in the genome of *Novopshingobium aromaticivorans* DSM 12444 [GenBank:NC_007794] and the bidirectional terminator BBa_B0014 [43]. *nicoTB* was codon-optimized for expression in *E. coli* using The Optimus [44] and the RCBSPC formula. Both genes were synthesized to contain the BioBrick prefix and suffix. *nicoTB* was then cloned downstream of *Ptac* by standard assembly, yielding the construct pIG49. *Ptac-nicoTB* (pIG49) was cloned upstream of *rcn*-*csgBAEFG* (pIG2, [22]) via a 3A assembly in pSB1T3, yielding pIG50.

### Visualization of biofilms using fluorescence microscopy

In each well of a 96-well plate, overnight cultures of fluorescent S29 (WT) and S61 (engineered) strains were diluted to an OD_600_ of 0.03 in a solution containing M63G medium, the appropriate antibiotic and metal at the appropriate concentration. Non-adherent bacteria were discarded after 1 hour of incubation at 30°C. M63G (0.2 mL) supplemented with the appropriate antibiotic and metal was then added to each well. The plates were then incubated at 30°C for 5 hours, after which the adherent bacteria were directly observed in the wells using a fluorescence inverted microscope (IX81, Olympus, Japan) operating at excitation and emission wavelengths of 488 nm and 507 nm, respectively. The cells were observed again in this way after a further 24-hour incubation.

### Visualization and quantification of biofilm thickness using confocal microscopy

In parallel with the fluorescence microscopy observations, the biofilm architecture and thickness was evaluated using a confocal microscope (LSM 510 META, Zeiss, Germany) operating at excitation and emission wavelengths of 488 nm and 507 nm, respectively. Images were then analyzed using ImageJ (National Institutes of Health, USA) and Imaris x64 (Bitplane, UK) software.

### Quantification of biofilms using spectrofluorimetry

S29 and S61 cells were grown at 30°C in 96-well plates in the presence of a range of Ni(II) or Co(II) concentrations (0, 0.05, 0.1, 0.3, 0.5, 1, 2 and 5 μM). The fluorescence intensity was measured using a Plate CHAMELEON™V fluorimeter (Hidex, Finland). Measurements were performed after 5- and 24-hour incubations in the presence or absence of the culture supernatant. Data were analyzed using MikroWin 2000 data reduction software (Mikrotek, Germany). Fluorescent intensities were converted to the number of cells/mm^2^ using standard calibration data established based on a viable S29 cell count.

### Metal sensitivity assay

Cultures in early stationary phase were diluted to concentrations of 10^8^, 10^7^, 10^6^, 10^5^,10^4^ and 10^3^ cells/mL. Five microliters of each culture were then spotted on plates containing M63G supplemented with metal ions at various concentrations (1, 10, and 20 μM) and incubated at 37°C for 48 h.

### Nickel uptake assay

The nickel uptake assay was adapted from [29]. Bacteria were cultured in LB medium to an OD_600_ of 0.6. Cells were harvested and washed once in phosphate buffer (50 mM NaH_2_PO_4_-Na_2_HPO_4_, pH 7.2). Bacteria were resuspended (1:5 v/v) in phosphate buffer supplemented with 0.2% glucose and 10 mM MgCl_2_. Transport was initiated by the addition of ^63^NiCl_2_ (Amersham biosciences, UK) at a final concentration of 150 nM. Bacterial cells were collected by filtration on nitrocellulose filters (0.45 μm pore size, Millipore, Germany) for the times indicated in Figure 3. The filters were washed twice with the same phosphate buffer, supplemented with 10 mM EDTA. Radioactivity was counted by using a Tri-Carb 2100TR scintillation counter (Canberra Packard, Austria) and the results were converted to pmole of Ni/mg of bacterial dry weight using an external standard curve.

### Metal titration using the ICP-MS technique

Overnight cultures were inoculated into 20 mL M63G medium supplemented with the appropriate antibiotic in Petri dishes. After 24 hours at 30°C, the supernatant was discarded, retaining the biofilm. To the biofilms, 10 mL of Ni(II) or Co(II) solutions (0, 5, 10, 12, 20 and 50 μM) were added. After a 10- (Figure 4A) or 30-minute contact period (Figure 4B and C), metallic supernatants were discarded and the biofilms were recovered. Each biofilm was scraped off into 1 mL of diluted M63 medium (1/4). The suspension was homogenized by vortexing for 20 s. To estimate the cell concentration in this biofilm fraction, 100 μL were removed for OD_600_ analysis. To the remaining biofilm suspension, 1.4 mL of 12 M nitric acid was added. Each sample was then incubated in a water bath at 80°C for 1 hour. Samples were stored at 4°C before analysis. For the ICP analysis, which was performed on a 7500cx mass spectrometer (Agilent, USA), the samples were diluted 100 times in a 0.5 M HNO_3_/2 μg/L rhodium solution. Rhodium was used as an internal standard.

## Competing interests

The authors declare that no competing interests exist.

## Authors’ contributions

AD, PL, VD, AR and CD conceived the study. AD, VC and CG constructed the strains and plasmids. AD, VC, FF, FS and CG performed the adherence experiments. AD, YL and AR performed the nickel uptake experiments. FF and YL performed the ICP-MS experiments. AD, FF, AR and CD prepared the manuscript. All authors read and approved the final manuscript. This work results from a wide collaboration of students and academics and stems from the iGEM “Cobalt Buster” project, which was conducted in 2011. Consequently, this study required much summer work by many students and instructors, justifying the need for 10 authors.

## References

[B1] Besser JM Leib KJ Church SE, von Guerard P, Finger SE Toxicity of metals in water and sediment to aquatic biota Integr Investig Environ Eff Hist Min Animas River Watershed S Juan Cty Colo 2007

[B2] Ahluwalia SS Goyal D Microbial and plant derived biomass for removal of heavy metals from wastewater Bioresour Technol 2007 8 2243 2257 10.1016/j.biortech.2005.12.006 16427277

[B3] Raghu G Balaji V Venkateswaran G Rodrigue A Maruthi Mohan P Bioremediation of trace cobalt from simulated spent decontamination solutions of nuclear power reactors using E. coli expressing NiCoT genes Appl Microbiol Biotechnol 2008 8 571 578 10.1007/s00253-008-1741-6 18949474

[B4] Mustafa YA Zaiter MJ Treatment of radioactive liquid waste (Co-60) by sorption on Zeolite Na-A prepared from Iraqi kaolin J Hazard Mater 2011 8 228 233 21955658 10.1016/j.jhazmat.2011.09.013

[B5] Volesky B Detoxification of metal-bearing effluents: biosorption for the next century Hydrometallurgy 2001 8 203 216 10.1016/S0304-386X(00)00160-2

[B6] Lloyd JR Bioremediation of metals; the application of micro-organisms that make and break minerals Interactions 2002 8 M2

[B7] Soares EV Soares HMVM Cleanup of industrial effluents containing heavy metals: a new opportunity of valorising the biomass produced by brewing industry Appl Microbiol Biotechnol 2013 8 6667 6675 10.1007/s00253-013-5063-y 23824444

[B8] Ruiz ON Alvarez D Gonzalez-Ruiz G Torres C Characterization of mercury bioremediation by transgenic bacteria expressing metallothionein and polyphosphate kinase BMC Biotechnol 2011 8 82 10.1186/1472-6750-11-82 21838857 PMC3180271

[B9] Macaskie LE Empson RM Cheetham AK Grey CP Skarnulis AJ Uranium bioaccumulation by a Citrobacter sp. as a result of enzymically mediated growth of polycrystalline HUO2PO4 Science 1992 8 782 784 10.1126/science.1496397 1496397

[B10] Roane TM Rensing C Peper IL Maier RM Maier RM, Pepper IL, Gerba CP Microorganism and metal pollutants Environmental Microbiology (2d edition), chapter 21 2009 Academic Press 421 441 http://www.sciencedirect.com/science/book/9780123705198

[B11] Kulkarni S Ballal A Apte SK Bioprecipitation of uranium from alkaline waste solutions using recombinant Deinococcus radiodurans J Hazard Mater 2013 8 853 861 24140537 10.1016/j.jhazmat.2013.09.057

[B12] Monier M Ayad DM Wei Y Sarhan AA Adsorption of Cu(II), Co(II), and Ni(II) ions by modified magnetic chitosan chelating resin J Hazard Mater 2010 8 962 970 10.1016/j.jhazmat.2010.01.012 20122793

[B13] Navarro C Wu L-F Mandrand-Berthelot M-A The nik operon of Escherichia coli encodes a periplasmic binding-protein-dependent transport system for nickel Mol Microbiol 1993 8 1181 1191 10.1111/j.1365-2958.1993.tb01247.x 7934931

[B14] Hebbeln P Eitinger T Heterologous production and characterization of bacterial nickel/cobalt permeases FEMS Microbiol Lett 2004 8 129 135 10.1016/S0378-1097(03)00885-1 14734175

[B15] Rodionov DA Hebbeln P Gelfand MS Eitinger T Comparative and functional genomic analysis of prokaryotic nickel and cobalt uptake transporters: evidence for a novel group of ATP-binding cassette transporters J Bacteriol 2006 8 317 327 10.1128/JB.188.1.317-327.2006 16352848 PMC1317602

[B16] Mulrooney SB Hausinger RP Nickel uptake and utilization by microorganisms FEMS Microbiol Rev 2003 8 239 261 10.1016/S0168-6445(03)00042-1 12829270

[B17] Deng X He J He N Comparative study on Ni2 + −affinity transport of nickel/cobalt permeases (NiCoTs) and the potential of recombinant Escherichia coli for Ni2+ bioaccumulation Bioresour Technol 2013 8 69 74 23306112 10.1016/j.biortech.2012.11.133

[B18] Zhang Y-M Yin H Ye J-S Peng H Zhang N Qin H-M Yang F He B-Y Cloning and expression of the nickel/cobalt transferase gene in E. coli BL21 and bioaccumulation of nickel ion by genetically engineered strain Huan Jing Ke Xue Huanjing Kexue Bian Ji Zhongguo Ke Xue Yuan Huan Jing Ke Xue Wei Yuan Hui Huan Jing Ke Xue Bian Ji Wei Yuan Hui 2007 8 918 923 17639961

[B19] Krishnaswamy R Wilson DB Construction and characterization of an escherichia coli strain genetically engineered for Ni(II) bioaccumulation Appl Environ Microbiol 2000 8 5383 5386 10.1128/AEM.66.12.5383-5386.2000 11097917 PMC92471

[B20] Rodrigue A Effantin G Mandrand-Berthelot M-A Identification of rcnA (yohM), a nickel and cobalt resistance gene in escherichia coli J Bacteriol 2005 8 2912 2916 10.1128/JB.187.8.2912-2916.2005 15805538 PMC1070376

[B21] Barnhart MM Chapman MR Curli biogenesis and function Annu Rev Microbiol 2006 8 131 147 10.1146/annurev.micro.60.080805.142106 16704339 PMC2838481

[B22] Drogue B Thomas P Balvay L Prigent-Combaret C Dorel C Engineering adherent bacteria by creating a single synthetic curli operon J Vis Exp JoVE 2012 e4176 http://www.jove.com/video/4176/engineering-adherent-bacteria-creating-single-synthetic-curli 10.3791/4176 PMC3523425 23183588

[B23] Blaha D Arous S Blériot C Dorel C Mandrand-Berthelot M-A Rodrigue A The Escherichia coli metallo-regulator RcnR represses rcnA and rcnR transcription through binding on a shared operator site: insights into regulatory specificity towards nickel and cobalt Biochimie 2011 8 434 439 10.1016/j.biochi.2010.10.016 21040754

[B24] Górecka E Jastrzębska M Immobilization techniques and biopolymer carriers Biotechnol Food Sci 2011 8 nr 1 65 86

[B25] Miao H Ratnasingam S Pu CS Desai MM Sze CC Dual fluorescence system for flow cytometric analysis of Escherichia coli transcriptional response in multi-species context J Microbiol Methods 2009 8 109 119 10.1016/j.mimet.2008.09.015 18926860

[B26] Perrin C Briandet R Jubelin G Lejeune P Mandrand-Berthelot M-A Rodrigue A Dorel C Nickel promotes biofilm formation by escherichia coli K-12 strains that produce curli Appl Environ Microbiol 2009 8 1723 1733 10.1128/AEM.02171-08 19168650 PMC2655473

[B27] pSB1C3 is a high copy number plasmid (RFC [10]) carrying chloramphenicol resistance [http://parts.igem.org/Part:pSB1C3?title=Part:pSB1C3]

[B28] pSB1T3 is a high copy number plasmid (RFC [10]) carrying tetracycline resistance [http://parts.igem.org/Part:pSB1T3?title=Part:pSB1T3]

[B29] Bleriot C Effantin G Lagarde F Mandrand-Berthelot M-A Rodrigue A RcnB is a periplasmic protein essential for maintaining intracellular Ni and Co concentrations in escherichia coli J Bacteriol 2011 8 3785 3793 10.1128/JB.05032-11 21665978 PMC3147525

[B30] Pesavento C Becker G Sommerfeldt N Possling A Tschowri N Mehlis A Hengge R Inverse regulatory coordination of motility and curli-mediated adhesion in Escherichia coli Genes Dev 2008 8 2434 2446 10.1101/gad.475808 18765794 PMC2532929

[B31] Jubelin G Vianney A Beloin C Ghigo J-M Lazzaroni J-C Lejeune P Dorel C CpxR/OmpR interplay regulates curli gene expression in response to osmolarity in Escherichia coli J Bacteriol 2005 8 2038 2049 10.1128/JB.187.6.2038-2049.2005 15743952 PMC1064031

[B32] Ogasawara H Yamamoto K Ishihama A Role of the biofilm master regulator CsgD in cross-regulation between biofilm formation and flagellar synthesis J Bacteriol 2011 8 2587 2597 10.1128/JB.01468-10 21421764 PMC3133154

[B33] Wasi S Tabrez S Ahmad M Toxicological effects of major environmental pollutants: an overview Environ Monit Assess 2013 8 2585 2593 10.1007/s10661-012-2732-8 22763655

[B34] Nair S Joshi-Saha A Singh S Ramachandran V Singh S Thorat V Kaushik CP Eapen S D’Souza SF Evaluation of transgenic tobacco plants expressing a bacterial Co–Ni transporter for acquisition of cobalt J Biotechnol 2012 8 422 428 10.1016/j.jbiotec.2012.07.191 22898176

[B35] Brim H McFarlan SC Fredrickson JK Minton KW Zhai M Wackett LP Daly MJ Engineering Deinococcus radiodurans for metal remediation in radioactive mixed waste environments Nat Biotechnol 2000 8 85 90 10.1038/71986 10625398

[B36] Rayu S Karpouzas DG Singh BK Emerging technologies in bioremediation: constraints and opportunities Biodegradation 2012 8 917 926 10.1007/s10532-012-9576-3 22836784

[B37] Lee J-C Pandey BD Bio-processing of solid wastes and secondary resources for metal extraction - a review Waste Manag 2012 8 3 18 10.1016/j.wasman.2011.08.010 21925857

[B38] Miller JH Experiment in Molecular Genetics 1972 Cold Spring Harbor, N.Y.: Cold Spring Harbor Laboratory Press

[B39] De Pina K Navarro C Mcwalter L Boxer DH Price NC Kelly SM Mandrand-Berthelot M-A Wu L-F Purification and characterization of the periplasmic nickel-binding protein NikA of escherichia coli K12 Eur J Biochem 1995 8 857 865 10.1111/j.1432-1033.1995.tb20211.x 7867647

[B40] Chung CT Niemela SL Miller RH One-step preparation of competent Escherichia coli: transformation and storage of bacterial cells in the same solution Proc Natl Acad Sci U S A 1989 8 2172 2175 10.1073/pnas.86.7.2172 2648393 PMC286873

[B41] Help: Assembly - parts.igem.org [http://parts.igem.org/Help:Assembly?title=Help:Assembly]

[B42] RBS [http://2009.igem.org/Team:Paris/Parts_RBS]

[B43] Part: BBa B0014 - parts.igem.org [http://parts.igem.org/Part:BBa_B0014]

[B44] The Optimus [http://gcat.davidson.edu/igem10/opt/opt_index.html]

